# A Nanoplasmonic-Based Biosensing Approach for Wide-Range and Highly Sensitive Detection of Chemicals

**DOI:** 10.3390/nano11081961

**Published:** 2021-07-30

**Authors:** Francesco Arcadio, Luigi Zeni, Aldo Minardo, Caterina Eramo, Stefania Di Ronza, Chiara Perri, Girolamo D’Agostino, Guido Chiaretti, Giovanni Porto, Nunzio Cennamo

**Affiliations:** 1 Department of Engineering, University of Campania Luigi Vanvitelli, Via Roma, 29, 81031 Aversa, Italy; francesco.arcadio@unicampania.it (F.A.); luigi.zeni@unicampania.it (L.Z.); aldo.minardo@unicampania.it (A.M.); caterina.eramo@unicampania.it (C.E.); stefania.dironza@unicampania.it (S.D.R.); chiara.perri@unicampania.it (C.P.); 2 Moresense Srl., Filarete Foundation, Viale Ortles 22/4, 20139 Milan, Italy; g.dagostino@moresense.tech (G.D.); g.chiaretti@moresense.tech (G.C.); g.porto@moresense.tech (G.P.)

**Keywords:** nanoplasmonic sensors, slab waveguides, plastic optical fibers, biochemical sensors, optical sensors, e-beam lithography

## Abstract

In a specific biosensing application, a nanoplasmonic sensor chip has been tested by an experimental setup based on an aluminum holder and two plastic optical fibers used to illuminate and collect the transmitted light. The studied plasmonic probe is based on gold nanograting, realized on the top of a Poly(methyl methacrylate) (PMMA) chip. The PMMA substrate could be considered as a transparent substrate and, in such a way, it has been already used in previous work. Alternatively, here it is regarded as a slab waveguide. In particular, we have deposited upon the slab surface, covered with a nanograting, a synthetic receptor specific for bovine serum albumin (BSA), to test the proposed biosensing approach. Exploiting this different experimental configuration, we have determined how the orientation of the nanostripes forming the grating pattern, with respect to the direction of the input light (longitudinal or orthogonal), influences the biosensing performances. For example, the best limit of detection (LOD) in the BSA detection that has been obtained is equal to 23 pM. Specifically, the longitudinal configuration is characterized by two observable plasmonic phenomena, each sensitive to a different BSA concentration range, ranging from pM to µM. This aspect plays a key role in several biochemical sensing applications, where a wide working range is required.

## 1. Introduction

Planar waveguide-based sensors have shown great potential in several application fields and represent an active research area. The development of these devices has mainly been boosted by the increasing need for rapid and automated devices to operate in several areas [1,2,3,4,5,6,7,8,9]. In particular, these kinds of platforms can also be used to exploit Surface Plasmon Resonance (SPR) and Localized Surface Plasmon Resonance (LSPR) phenomena in order to achieve excellent performances in biosensing applications [10,11,12,13,14,15,16,17].

In the scientific literature, several configurations exist, based on planar slab waveguide integrated on silicon (Si)-based substrate used for multiple applications, like gas sensing [18,19,20,21,22], magnetic field sensing [23,24], biological and chemical species detection [2,6,25,26,27], and so on. These platforms take advantage of the technological and fabrication processes of the microelectronic industry. However, the main disadvantages of this approach are related to the difficulties connected to those techniques in realizing thick layers, despite the several efficient solutions that have been proposed during the last years [28]. Moreover, when planar slab waveguides based on silicon are applied, the technology used to realize them could be very expensive, especially in prototype realization. More specifically, when considering plasmonic sensors, the number of modes (depending on the guiding layer thickness) propagating into the waveguide plays a fundamental role in the performance of the conceived sensor. In particular, in the last years, multimodal waveguides have been preferred because, even if they usually show a non-optimum signal to noise ratio (SNR), they present a better sensitivity than monomodal waveguides [29,30]. This aspect is crucial if related to biochemical sensing applications, where high sensitivity is required to detect as low a concentration as possible of the analyte of interest.

In any case, the trade-off between SNR and sensitivity has been largely investigated and it is one of the reasons why, for instance, large core diameter plastic optical fibers (POFs) are preferred instead of monomodal silica ones when realizing SPR sensors based on optical fibers [31,32,33,34]. So, multimode polymer slab waveguides have also drawn the scientific community’s attention so far, showing excellent performances and results [7,14,35,36].

We exploited a multimode slab waveguide based on a Poly(methyl methacrylate) substrate to realize a nanoplasmonic biosensor. This PMMA chip has already shown its capability to be used as a plasmonic sensor, as reported in [14]. In particular, the PMMA chip is able to ensure an appropriate number of angles that satisfy the SPR condition at the interface between the metallic layer and the dielectric medium [14]. For this reason, this SPR probe has also been successfully tested in chemical analysis, like in the furfural detection by a specific biomimetic receptor [37]. Despite this, the overall performances of the SPR sensor based on the PMMA slab waveguide are similar to an SPR D-shaped POF sensor [38]. Actually, the most valuable advantage in its usage is mainly related to the very simple fabrication process that includes only a 60 nm thick gold deposition step upon the PMMA substrate [14].

In order to exploit the maximum potential of this type of plastic slab waveguide chip and take full advantage of its dimension (10 mm × 10 mm × 0.5 mm), fully compliant to the modern holder of electron beam lithography (EBL) systems (e.g., Zeiss Supra v35—Raith Elphy Quantum system), we conceived and tested a plasmonic sensor based on a gold nanograting (GNG) fabricated on the top of this PMMA chip [39]. In particular, as proof of concept, we functionalized the nano groove surface with a synthetic receptor specific for the bovine serum albumin (BSA), and we tested its biosensing capabilities by using an experimental setup described in [39]. In particular, in [39] the PMMA substrate has been considered a transparent substrate instead of a waveguide. Moreover, a similar nanostructure has also demonstrated an excellent solution to realize Surface-enhanced Raman scattering (SERS) active substrates [40].

Interesting work has described the excellent progress in developing novel surface functionalization strategies and the formation of optically and mechanically stable LSPR sensors [41].

In this work, we used a different approach by considering the PMMA chip not like a transparent substrate but as a slab waveguide to excite the plasmonic phenomena. We functionalized the nanograting surface with a synthetic receptor specific for BSA to compare the sensor performances with other sensor configurations. In such a way, we first established how, in this sensor configuration, the nanostripes’ orientation with respect to the direction of the input light beam (longitudinal or orthogonal) influences the biosensing capabilities. Moreover, we established that two distinct plasmonic phenomena can widen out the operating range relative to the BSA detection when considering the longitudinal configuration. The proposed experimental setup includes an aluminum holder that contains the PMMA-GNG chip and two POF patches used to connect the holder with a white light source and a spectrometer. Finally, we also carried out a comparative analysis in terms of limit of detection (LOD) with an SPR probe based on POF and functionalized with the same receptor. In this work, exploiting the proposed setup, we changed the angle of the incident light with respect to [39] in the plasmonic phenomena obtaining different performances. In particular, we used the best configuration obtained in [39] in terms of geometric parameters of the GNG. We do not present any parametric optimization of the device in numerical and experimental results, as reported in [39], even if the geometric parameter variation influences the performances. In fact, this work aims to compare the performances of the proposed setup with those proposed in [39].

## 2. Materials and Methods

### 2.1. Plasmonic Sensor Fabrication

To compare the results with those obtained exploiting a different sensor configuration [39], we used the same nanoplasmonic chip reported in [39].

In particular, each stripe presents a width equal to about 400 nm, whereas the mutual spacing is equal to about 615 nm, which means a grating period equal to about 1 µm.

**Figure 1:**
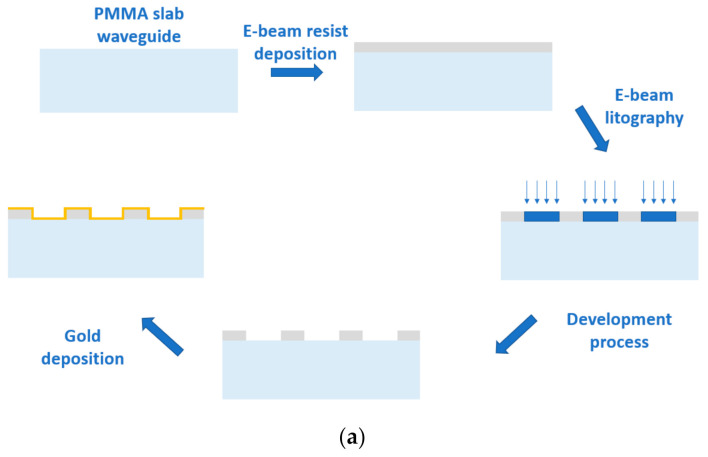

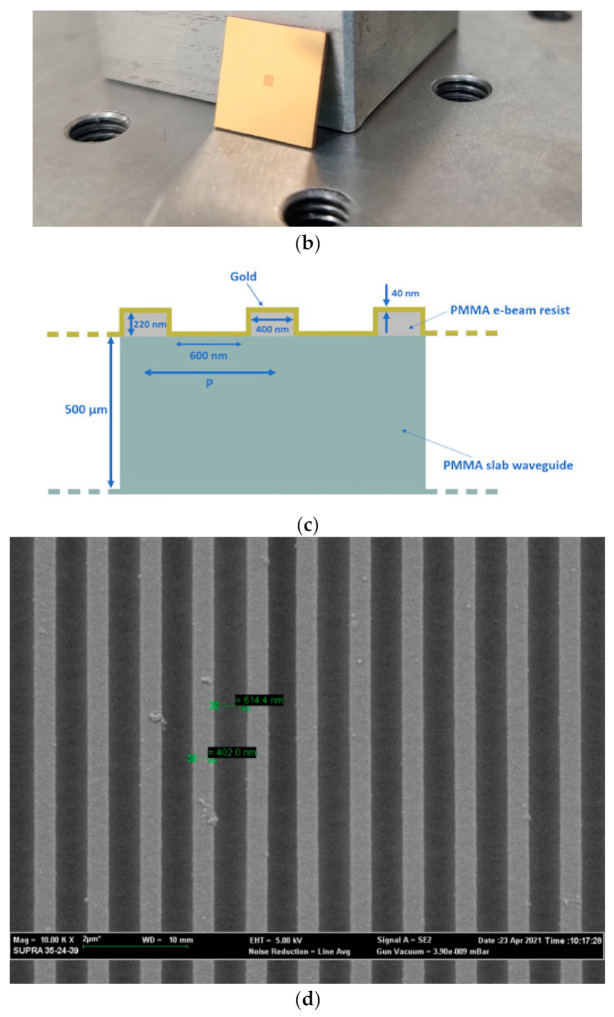
(**a**) Outline of the fabrication process of the plasmonic sensor. (**b**) picture of the PMMA slab waveguide with gold nanograting pattern at the center. (**c**) Schematic cross section of the examined plasmonic GNG-based chip with the relative dimensions. (**d**) SEM image of the fabricated nanograting.

The device fabrication steps are schematically recalled in Figure 1a. In summary, to realize the nanoplasmonic chip, the starting sample consists of a 10 mm × 10 mm × 0.5 mm PMMA slab waveguide (GoodFellow, Huntingdon, England) where, on its surface, a positive PMMA e-beam resist (AR-P 679.04, AllResist GmbH, Strausberg, Germany) is deposited through a spin coater running at 6000 rpm for one minute (a film with a final thickness of about 220 nm). The exposition process, carried out by using an electron beam lithography system (Zeiss Supra v35—Raith Elphy Quantum, Oberkochen, Germany), takes place in a total area of 1 mm^2^ (1 mm × 1 mm) at the center of the slab waveguide (see Figure 1b). The final steps consist of developing the exposed resist and depositing a 40 nm thick gold film through a sputtering machine (BalTec SCD 500, Schalksmühle, Germany). The cross section of the realized plasmonic GNG-based chip with the relative dimensions is shown in Figure 1c,d reports a Scanning Electron Microscope (SEM) image of the fabricated nanograting.

In this work, we did not carry out atomic force microscopy images and topographic profiles of the deposited gold film. So, even if the roughness of the gold film influences the sensitivity of the plasmonic sensor, we could not consider this aspect to modify the sensor performances. In fact, this work focuses on comparing the proposed sensor configuration and that used in [39]. So, we took care to realize the plasmonic chip in a similar way to that used in [39], as has been described in this section.

### 2.2. Synthetic Receptor Film

#### 2.2.1. Chemicals

Reagents: *N*,*N*’-methylene bisacrylamide (BIS) (CAS 110-26-9), Acrylamide (Aam) (CAS 79-06-1), *N*-tert-butylacrylamide (TBAm) (CAS 107-58-4), 2-hydroxyethyl methacrylate(HEMA) (CAS 868-77-9), ammonium persulfate (APS) (CAS 7727-54-0), *N*,*N*,*N*’,*N*’-tetramethylethylenediamine (TEMED) (CAS 110-18-9, sodium dodecyl sulfate (SDS) (CAS 151-21-3), phosphate buffer solution 1.0 M were from Sigma-Aldrich (Darmstadt, Germany) and used without any further purification. All other chemicals were of analytical reagent grade. The solvent was Milli-Q water.

The bovine serum albumin (CAS 9048-46-8) and trypsin (CAS 9002-07-7) were from Sigma-Aldrich (Darmstadt, Germany).

#### 2.2.2. Molecularly Imprinted Polymer for BSA Detection

Molecularly imprinted polymers (MIPs) are synthetic receptors able to recognize specific molecules or a class of molecules [42,43]. The synthesis is based on using a template molecule and appropriate functional monomers, which coordinate the target molecule by establishing interactions of various kinds (van-der Waals, ionic, dipole-dipole, etc.), forming a complex.

Subsequently, a cross-linking reagent fixes the complex, forming a polymer around it. At the end of the polymerization process, the template is removed, leaving the interaction sites free and able to reversibly recognize the analyte of interest.

In this study, a synthetic receptor was grown on the Au surface in nanometric film. In particular, a recently developed molecular imprinted polymer synthesis strategy for proteins, under non-denaturing conditions, is used [44]. The preparation is reported below.

With the purpose of covalently binding the polymeric receptor to the gold layer, the optical transducer was first modified with an allyl thiol. In particular, the gold surface of the transducer was immersed in a 10% *v*/*v* solution of allyl thiol in 80% *v*/*v* ethanol solution and 10% *v*/*v* water for 12 h. Subsequently, the platform was washed with Milli-Q water (flushing 3 mL 5 times). This process formed a self-assembled monolayer with a terminal allyl group.

The monomer mixture was prepared by adding Acrylamide (Aam), *N*-t-butylacrylamide (TBAm), 2-hydroxyethyl methacrylate (HEMA) at 1:0.5:0.6 molar ratio, in a 15-mM phosphate buffer (PB) pH 7.4. Then, *N*,*N*’-methylene bisacrylamide (BIS) was added to the monomeric mix with a final concentration of 0.19 M. The pre-polymeric mixture was dispersed by sonication (sonic bath model VWR USC200T) for 10 min and bubbled with N2 for 30 min at room temperature. The template (BSA) was added to the pre-polymeric mixture to the final concentration of 1 μM. Then APS (0.08% *w*/*v*) and TEMED (0.06% *w*/*v*) were added. About 2 µL of the pre-polymeric mixture was dropped over the sensing region and let polymerize for 15 min at room temperature, after which the reticulation process was stopped by washing the sensor surface with Milli-Q water. Finally, the template was removed by incubating trypsin 4.2 × 10^−8^ M for 2 h at room temperature on the sensor surface and then by washing with an SDS 5% (*w*/*v*) solution. We used an MIP for BSA to compare the results obtained in this work with those carried out in [42,43].

### 2.3. Experimental Setup

In this work, to monitor the GNG-based sensor configuration, we used an experimental setup that exploits the PMMA substrate, acting as a slab waveguide, to excite the nanoplasmonic phenomenon. It consists of a white light source (HL-2000-LL, manufactured by Ocean Optics, Dunedin, FL, USA, with an emission range from 360 nm to 1700 nm), a spectrometer (FLAME-S-VIS-NIR-ES, manufactured by Ocean Optics, Dunedin, FL, USA, with a detection range from 350 nm to 1023 nm), two POF patches, and an aluminum holder. We did not control the light’s polarization in this setup because we used a multimode optical waveguide and a trench of air to couple the light in the waveguide.

**Figure 2:**
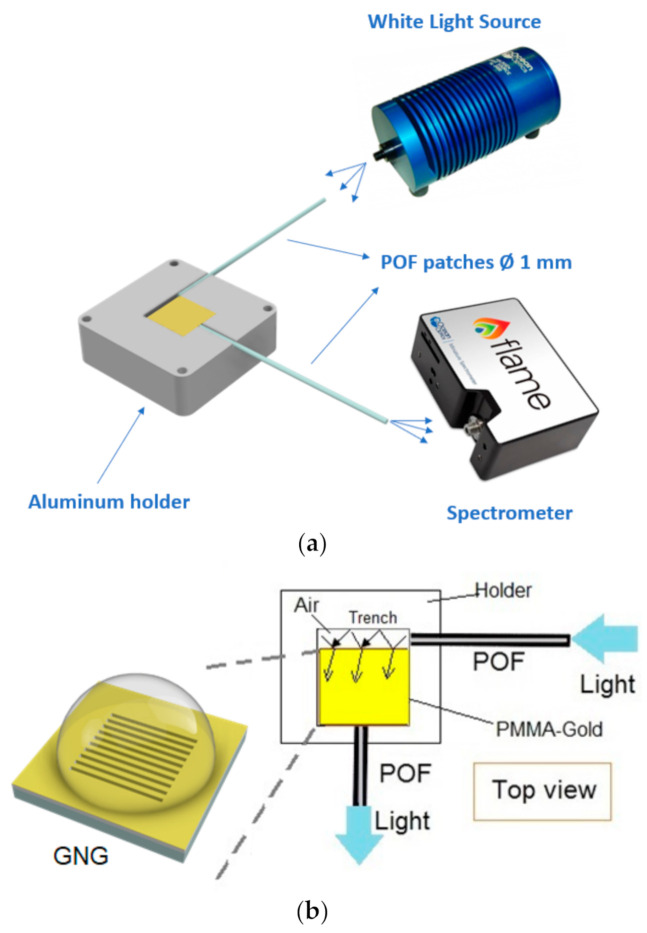
(**a**) Outline of the experimental setup. (**b**) Light propagation path through the setup.

As shown in Figure 2a, all these components are connected similarly to SPR sensors [14]. Figure 2b shows the light propagation path through the setup. In particular, the light is launched from the source to a first POF patch (1 mm total diameter). At the end of the POF, a trench of air is realized in the metallic holder, which can be used to enlarge the number of angles useful to excite plasmons in the nanostructured slab waveguide of PMMA-gold. On the other hand, another POF patch (1 mm total diameter) kept at the end of the PMMA-gold waveguide, at a 90° angle with respect to the air trench, collects the transmitted light through nanostructured slab waveguide of PMMA-gold to direct it towards the spectrometer.

## 3. Results

In the considered setup, in the nano-patterned slab waveguide covered by gold nanofilm and MIP receptor layer, two different plasmonic phenomena, on the sensor surfaces interested in the functionalization process, can be excited.

**Figure 3:**
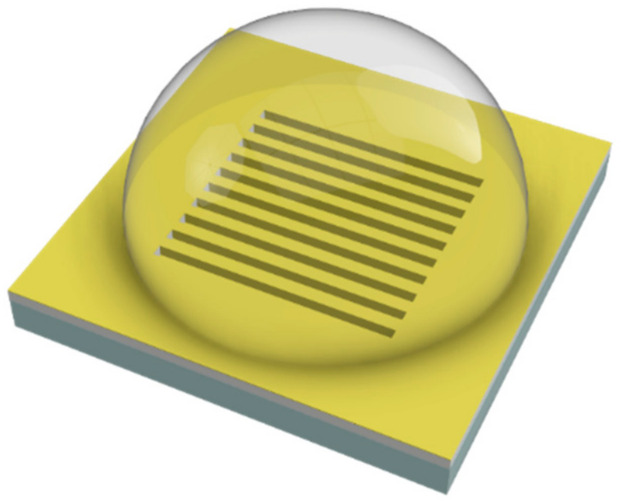
Outline of the functionalization process: the pre-polymeric mixture is dropped on the sensor surface and covers both the nanograting and the nearby regions.

We deposited the biomimetic receptor on a region larger than the GNG area (1 mm^2^). So, we had a receptor layer on the GNG surface and the nearby regions, where a continuous gold film was present, as schematically shown in Figure 3. Therefore, when considering the GNG structures, as already underlined in [39], plasmonic hybrid modes are excited due to the mutual interaction between the surface plasmons (SPs) and the localized surface plasmons (LSPs). In contrast, the classic SPR phenomenon occurs where the continuous gold film is present.

In Ref. [39], there was evidence only of the first kind of plasmonic phenomenon (hybrid modes) since the direction of the input light was orthogonal with respect to the sensor surface, and this working condition does not allow the excitation of SPs on the continuous gold film nearby the nanograting.

Moreover, when interrogating the same plasmonic GNG-based sensor using the experimental setup reported in [39], neither the resonance wavelength areas nor the optical performances were influenced by the nanostripes’ orientation with respect to the direction of the input light.

**Figure 4:**
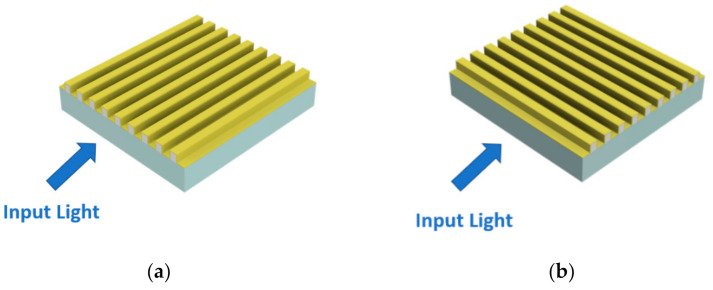
(**a**) “GNG-based longitudinal” configuration. (**b**) “GNG-based orthogonal” configuration.

In contrast to the above, we determined that these aspects have particular relevance in the sensor configurations proposed here. For this reason, as schematically shown in Figure 4, we identified two possible sensor configurations, that is, “GNG-based longitudinal”, where the nanostripes are located along the same direction of the input light (see Figure 4a), and “GNG-based orthogonal”, when the two mentioned directions are mutually orthogonal (see Figure 4b).

**Figure 5:**
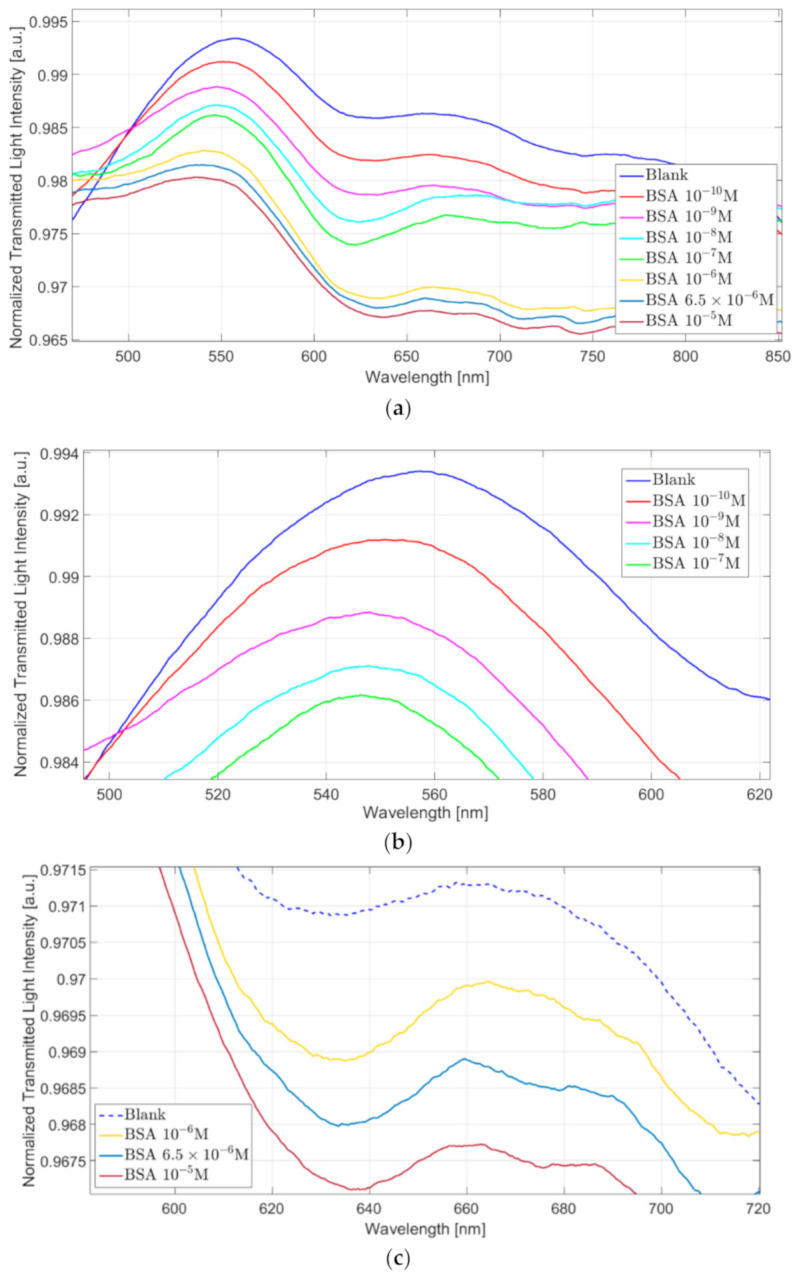
(**a**) Plasmonic spectra at varying of the BSA concentration (0–10^−5^ M) relative to “GNG-based longitudinal” configuration. Zoom of the resonance wavelength area relative to (**b**) peak at 550 nm and (**c**) peak at 630 nm.

Figure 5a reports the plasmonic spectra relative to the “GNG-based longitudinal” configuration at different BSA concentrations. These spectra were obtained by normalizing the transmitted spectra to the one obtained with air as the surrounding medium. This kind of normalization was possible in this setup because we had no evidence of resonance in air, in contrast to what is shown in [39]. As shown in the zooms of Figure 5, reported in Figure 5b,c, two distinct resonance phenomena can be observed at around 550 nm (plasmonic hybrid modes) and 630 nm (SPR).

In the considered case, by analyzing more in detail these two plasmonic resonances, it is clear that they are sensitive to a different range of BSA concentrations.

In particular, at low concentrations (10^−10^ M–10^−7^ M), the resonance wavelength peak at 550 nm decreases (blue shift) when the BSA concentration increases, as shown in Figure 5b. On the opposite, in a different BSA range (10^−6^ M–10^−5^ M) the resonance wavelength peak at 630 nm increases (red shift) when the analyte concentration increases too (see Figure 5c). The first behavior (blue shift at around 500 nm) is similar to the one observed in [39]. It can be ascribed to the hybrid modes excited by the nanograting, even if at different resonance conditions. The second one (red shift at around 650 nm) is typical when considering SPR phenomenon.

**Figure 6:**
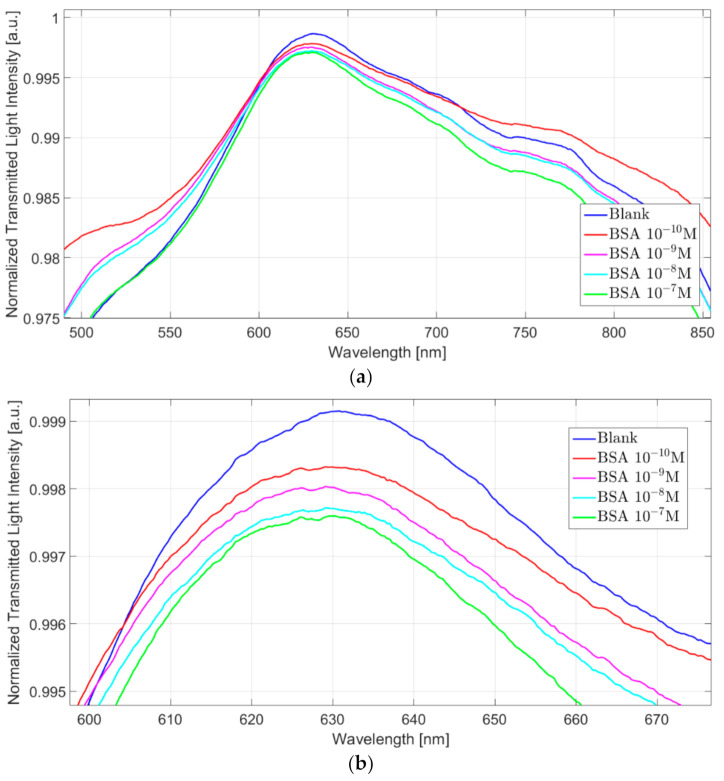
(**a**) Plasmonic spectra at varying of the BSA concentration (0–10^−7^ M) relative to “GNG-based orthogonal” configuration. (**b**) Zoom of the resonance wavelength area.

Conversely, when considering the “GNG-based orthogonal” configuration, only a resonance peak at 630 nm is visible, as shown in Figure 6a. In particular, at increasing BSA concentration, the resonance wavelength decreases similarly to what we observed with the resonance peak at 550 nm in the “GNG-based longitudinal” configuration. Figure 6b shows a zoom of the resonance wavelength region.

This aspect could be explained by the fact that a 90-degree-rotation of the pattern causes a wavelength overlap of the two previously mentioned plasmonic phenomena. In fact, in our previous work [45], we demonstrated, by performing numerical simulations in a similar structure, that when considering an orthogonal configuration, the GNG’s resonance wavelength area shifts towards higher values with respect to the longitudinal one [45].

The observed resonance could be ascribable to the GNG (i.e., the blue-shift one) because it is predominant over the other one, even if it is negatively influenced by the SPR phenomenon, which goes in the opposite direction. This mutual destructive interference between these different plasmonic phenomena (blue shift and red shift) is confirmed by a worse optical sensitivity. The orthogonal configuration denotes a minor total shift in resonance wavelength between the blank and higher BSA concentrations.

## 4. Discussion

**Figure 7:**
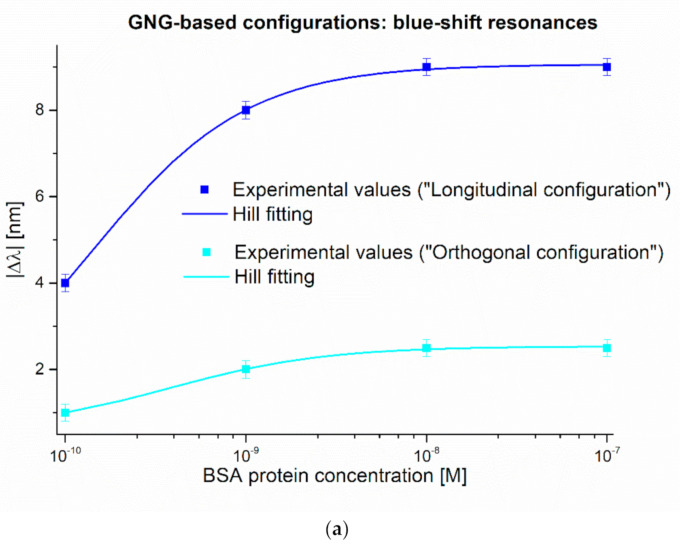

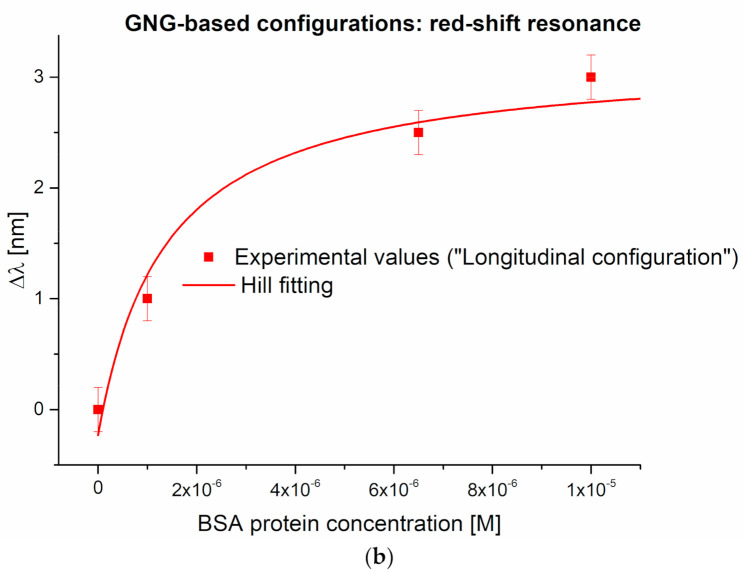
BSA dose-response curves with Hill fitting of the data and error bars relative to (**a**) GNG-based longitudinal and GNG-based orthogonal blue-shift resonances and (**b**) GNG-based longitudinal red-shift resonance.

Figure 7 reports the dose-response curves carried out for the BSA binding test measurements, along with the Hill fitting of the experimental values and the error bars, for both the analyzed configurations (longitudinal and orthogonal). In particular, Figure 7a shows the BSA detection relative to the blue shift resonances, whereas the detection carried out by the red shift resonance is reported in Figure 7b. In Figure 7, each experimental value was calculated as the absolute value variation with respect to the blank solution (i.e., a solution without the analyte). The Hill equation used to fit the experimental data is reported as follows:

(1) $$\left|{\Delta \lambda }_{c}\right|=\left|\lambda_{c}-{\;\lambda }_{0}\right|=\;{\Delta \lambda }_{max}|\cdot \left(c^{n}/\left(K^{n}+{\;c}^{n}\right)\right)$$

where c is the analyte concentration, λ_c_ is the resonance wavelength at the concentration c, λ_0_ is the resonance wavelength at zero concentration (blank), ∆λ_max_ is the maximum value of ∆λ_c_ (calculated by the saturation value minus the blank value, i.e., λ_max_ ─ λ_0_). The parameters *n* and *K* are the Hill constants.

Table 1 reports the parameters relative to Equation (1) for each analyzed sensor configuration and each plasmonic phenomenon. Table 2 reports the chemical parameters calculated according to Equation (1) and considering *n* = 1 (in this case, the Hill equation is the same as the Langmuir equation). At low concentration, which means *c* much lower than *K*, Equation (1) can be approximated to a linear function, where the slope (Δλ_max_/K) is called “sensitivity at low concentration”. It is essential to underline that, to compare the limit of detection (LOD) with other SPR POF-based sensor configurations, we used the same LOD definition adopted in [39,44]. So, by considering the linear behavior at low BSA concentration, the latter is used to calculate the LOD defined as:

(2) LOD= 2·σλ0ΔλmaxK 

where $\sigma_{\lambda_{0}}$ represents the standard error relative to the blank solution, that is, the standard error relative to λ_0_ reported in Table 1. In Table 2, the sensitivity at low concentration, the affinity constant (Kaff), and the LOD are reported for the tested sensor configurations and the different plasmonic phenomena.

Finally, a comparative analysis relative to the BSA detection was reported in Table 3 regarding the LOD and the detection range. We compared these results with the ones reported in [39,44], where the same MIP receptor was used.

As it can be seen, we first confirmed that nanostructured sensors’ use leads to a significant improvement when considering biosensing applications, as already underlined in [39]. Moreover, we demonstrated that when we use the PMMA as a slab waveguide or as a transparent substrate, the biosensing performances at very low analyte concentrations (pM–nM) are similar. We obtained a similar LOD in both the analyzed configurations (longitudinal and orthogonal) with respect to the one obtained in [39]. The advantage of using the sensing approach adopted in this work is the possibility to monitor a wide range of analyte concentrations using a single plasmonic sensor. In fact, by exploiting the “GNG-based longitudinal” configuration, we widened the detection range by taking advantage of two distinct plasmonic phenomena. In particular, the blue shift resonance is sensitive in the BSA range between 23 pM (LOD) and 10 nM (saturation value). In contrast, the red-shift resonance is sensitive in the BSA range between 0.54 µM (LOD) and 10 µM (saturation value). These values, relative to the red-shift resonance, are similar to those obtained with the SPR-POF platform [44], where an LOD equal to 0.37 µM was obtained.

## 5. Conclusions

A specific experimental setup has been used to monitor a nanoplasmonic slab waveguide functionalized with a synthetic receptor for BSA detection. In particular, we have determined the influence of the nanostripes’ orientation with respect to the input light direction by identifying two possible sensor configurations, orthogonal and longitudinal. Both configurations have shown blue-shift resonances at increasing the target analyte concentrations, obtaining a similar LOD (≈pM) if compared to the one obtained with the same chip interrogated with a different experimental setup [39]. In addition, the longitudinal configuration has also denoted a red-shift resonance, ascribable to an SPR phenomenon, sensitive to higher BSA concentrations. In the latter case, exploiting the SPR phenomenon, we obtained an LOD (≅0.57 µM) similar to the one obtained with an SPR-POF reference sensor functionalized with the same receptor [44]. Standing this, the more evident advantage of the proposed sensing approach is the feasibility of monitoring a wide range of analyte concentrations (from pM to µM) by a single plasmonic platform (longitudinal configuration).

As a final remark, it is essential to underline that in this kind of experimental setup, a greatly reduced volume of solution (about 10 µL) is required, compared to the 1 mL used in the setup described in [39].

## Figures and Tables

**Table 1:** Hill fitting parameters relative to Equation (1) for different sensor configurations.

Configuration	λ_0_ (nm)		λ_max_ (nm)		K (M)		*n*	Statistics	
	Value	Stand. Err.	Value	Stand. Err.	Value	Stand. Err.	Value	Reduced Chi-Sqr	Adj. R-Square
GNG-based longitudinal (blue shift)	0.30	0.73	9.06	0.06	1.37 × 10^−10^	2.65 × 10^−11^	1	0.14	0.99
GNG-based orthogonal (blue shift)	0.59	0.11	2.54	0.03	3.74 × 10^−10^	7.56 × 10^−11^	1	0.05	0.99
GNG-based longitudinal (red shift)	−0.23	0.69	3.18	0.35	1.35 × 10^−6^	6.57× 10^−7^	1	1.17	0.98

**Table 2:** Chemical parameters relative to the BSA detection for the tested sensor configurations.

Configuration		Parameter		
	Plasmonic Phenomenon	Sensitivity at Low Concentration (nm/M)	LOD [M]	K_aff_ = 1/K (M^−1^)
GNG-based longitudinal (blue shift)	Hybrid	6.39 × 10^10^	2.3 × 10^−11^	7.3 × 10^9^
GNG-based orthogonal (blue shift)	Hybrid	5.2 × 10^9^	4.2 × 10^−11^	2.7 × 10^9^
GNG-based longitudinal (red shift)	SPR	2.53 × 10^6^	5.4 × 10^−7^	7.4 × 10^5^

**Table 3:** Comparative analysis in terms of limit of detection and BSA detection range for several plasmonic configurations combined with the same MIP receptor.

Configuration	LOD	BSA Detection Range	Reference
Gold nanograting on a PMMA substrate (same chip of this work in a different setup)	37 pM	37 pM–100 nM	[39]
SPR D-shaped POFs	0.37 µM	0.37 µM–6.5 µM	[44]
GNG-based longitudinal (blue-shift resonance)	23 pM	23 pM – 10 nM	This work
GNG-based longitudinal (red-shift resonance)	0.54 µM	0.54 µM–10 µM	This work
GNG-based orthogonal (blue-shift resonance)	42 pM	42 pM–10 nM	This work

## Data Availability

The data is available on reasonable request from the corresponding author.
